# Does hourly variation in ambient nitrogen dioxide increase the risk of acute asthma hospitalisations? A time-series study of Birmingham and Solihull, UK

**DOI:** 10.1136/bmjopen-2025-110612

**Published:** 2026-08-07

**Authors:** Nidhi Shukla, Alan Kwok, Alexander Topham, Suzy Gallier, Elizabeth Sapey, Andrew Percy, Mark Bayliss, Prasad Nagakumar, Anna L Hansell, Suzanne E Bartington, Tim CD Lucas

**Affiliations:** 1 Division of Public Health and Epidemiology, School of Medical Sciences, University of Leicester, Leicester, UK; 2 University of Leicester Centre for Environmental Health and Sustainability, Leicester, UK; 3 PIONEER HDR UK Hub, University Hospitals Birmingham NHS Foundation Trust, Birmingham, UK; 4 Department of Inflammation and Ageing, University of Birmingham, Birmingham, UK; 5 NIHR Birmingham Biomedical Research Centre, University of Birmingham, Birmingham, UK; 6 National Institute of Health Research (NIHR) Health Protection Research Unit (HPRU) in Chemical Threats and Hazards, University of Leicester, Leicester, UK; 7 National Institute of Health Research (NIHR) Leicester Biomedical Research Centre (BRC), Leicester General Hospital, Gwendolen Road, Leicester, UK; 8 Department of Applied Health Sciences, University of Birmingham, Birmingham, UK

**Keywords:** Health Impact Assessment, EPIDEMIOLOGIC STUDIES, Asthma, Risk Assessment, Risk Factors

## Abstract

**Abstract:**

**Objective:**

This study aims to investigate the association between hourly nitrogen dioxide (NO_2_) concentrations and emergency hospital admissions for adult patients with asthma in the Birmingham–Solihull metropolitan area, West Midlands, UK.

**Design:**

A time-series study.

**Setting:**

The study was conducted in the Birmingham–Solihull metropolitan area, West Midlands, UK.

**Participants:**

Adult patients with asthma (aged ≥16 years at the time of admission) from 1 June 2016 to 31 May 2022 residing in Birmingham and Solihull, West Midlands.

**Outcome measures:**

We analysed the hourly rate of emergency hospital admissions for acute asthma. A Poisson generalised additive model combined with the distributed lag non-linear model was applied to assess the association between hourly NO_2_ concentrations and hourly counts of hospital admissions for acute asthma. Furthermore, the effect modification of the NO_2_-asthma association in specific participant groups, such as sex, deprivation index and ethnicity, was explored in stratified analyses.

**Results:**

The study included 18 943 adults with asthma exacerbations who attended the four acute hospitals in the study area during the study period. The study observed a significant positive association between hospital admissions for acute asthma and hourly NO_2_ concentrations. The mean NO_2_ exposure (18 µg/m³) over a lag of 0–24 hours was associated with a 13% (RR=1.13, 95% CI 1.01 to 1.26) increase in the risk of daytime emergency hospital admissions for acute asthma in comparison with no exposure. The results show a significant association between NO_2_ exposure and hospital admissions for a lag of 3–6 hours. Furthermore, subgroup-specific analysis indicated a higher positive risk associated with hourly NO_2_ among patients living in the most deprived areas.

**Conclusions:**

This study strengthens the evidence for the importance of using high-temporal resolution air pollution data to examine impacts on health, particularly where there are marked temporal patterns in exposure. Understanding these exposure–response relationships will improve healthcare preparedness and resource allocation in relation to air pollution levels.

**STRENGTHS AND LIMITATIONS OF THIS STUDY**

- This study used exposure at a high-temporal hourly time scale.
- This study advances prior work by analysing the complete hourly nitrogen dioxide data rather than relying on time-stratified methods.
- The use of fixed-site monitors may have introduced exposure misclassification.
- This two-city study may limit the wider generalisation of its findings.
- The sample size was limited for specific subgroups.

## Introduction and background

 Asthma, a chronic inflammatory disease of the lungs, is prevalent across all age groups within populations. Its symptoms include coughing, wheezing, shortness of breath and chest tightness, varying in severity from mild to severe. Asthma affects over 262 million (224–309 million) people globally in 2019, and the UK experiences a high burden of morbidity and mortality associated with the condition, with approximately three deaths per day and 60 000 emergency hospital admissions annually.^1 2^ Asthma results in a significant but preventable healthcare and economic burden, including 200 000 bed days per year in the UK and £1.1 billion in annual National Health Service (NHS) costs.^3^ Asthma shows significant variability in prevalence and incidence based on age and gender. Although asthma is more common in children, adults experience higher rates of morbidity and mortality from the condition, reflecting complex interactions between different risk factors.^4^ Along with other triggers, depending on the asthma phenotype, environmental exposures to temperature and air pollution are major risk factors of asthma exacerbation.^5 6^

A meta-analysis of 84 studies showed a significant association between ambient air pollutants (fine particulate matter (PM_2.5_), PM_10_, carbon monoxide (CO), ozone (O_3_) and nitrogen dioxide (NO_2_)) and an increased risk of asthma exacerbations in both single day-0 and day-1 lag exposure patterns.^7^ A study demonstrated that asthmatic adults experience a decline in lung function after walking for 2 hours along the streets of London due to traffic-related pollution.^8^ While this study focused on PM_2.5_, NO_2_ is another key pollutant formed during combustion processes, with major sources, including road transport and industrial combustion. 30% nitrogen oxide (NOx) emissions, which include nitric oxide and NO_2_, were contributed by road transport in 2022 in the UK. The overall traffic volume in urban areas is closely related to NOx levels. Therefore, urban NO_2_ concentrations typically exhibit diurnal peaks due to changes in traffic activity during peak travel periods, thus making it crucial to use high-temporal-resolution hourly NO_2_ levels while accounting for the association between its exposures and respective risks of asthma exacerbation.^9 10^

NOx increases reactive oxygen species production in patients with asthma, leading to inflammation and reduced lung function.^11^ This effect is particularly pronounced in severe asthma cases. Furthermore, the literature provides strong biological evidence of an association between short-term hourly NO_2_ exposure and the exacerbation of asthma symptoms. Experimental studies on mice exposed to NO_2_ (5 ppm, 4 hours per day for 30 days) can induce airway inflammation, oxidative stress and promote a Th1/Th2 immune response, all of which are central to asthma pathophysiology.^12^ A controlled human exposure study demonstrated that exposure to traffic-related air pollutants such as NO_2_ during a 1.5-hour car ride in rush-hour traffic caused immediate increases in biomarkers of oxidative stress in the respiratory tract.^13^

The literature highlights the significance of both spatial and temporal high-resolution air pollution levels to assess the impact of air pollution on patients with asthma. A time-stratified case-crossover analysis conducted in China found that emergency department visits for asthma were highest during the hour of exposure (0 hour lag), rapidly declined over the following 3–12 hours, and weakened further thereafter.^14^ However, there are limited studies that use the complete temporal pattern of NO_2_ exposure, which exhibits a pronounced diurnal variation, and its association with asthma exacerbation. The previous evidence has indicated its association with emergency hospital admissions for asthma, although most studies have used low temporal resolution data, such as daily or monthly and time-stratified case-crossover analysis.^14 15^

Furthermore, a reduction in NO_2_ has been associated with a decline in the requirement for healthcare services. During the COVID-19 pandemic, widespread lockdowns led to a significant reduction in NO_2_ concentration.^16^ Studies have reported an association between the decline in NO_2_ concentration and adult asthma hospital admissions during 2020 compared with the previous years.^15^ Given that these pollutants have a strong diurnal pattern with specific spikes in level during peak hours, investigating the association between high-temporal-resolution NO_2_ measurements and hourly hospital admissions is critical to guide the informed development of targeted interventions that address diurnal patterns in NO_2_ concentrations.

This study examines the association between high temporal changes in NO_2_ concentrations and emergency hospital admissions for adult patients with acute asthma living in the Birmingham city and Solihull local authority areas within the West Midlands, UK. The West Midlands is a metropolitan UK region with recognised air quality challenges, with all residents living in locations estimated to exceed WHO Global Air Quality guidelines for annual average NO_2_ concentrations (10 µg/m³) in 2019.^17^ While previous literature has primarily focused on children, this study focuses on adults (aged ≥16 years), considering the available evidence showing a positive association between NO_2_ exposure and adult asthma.^18^ This study estimates the cumulative effect of 0-hour to 24-hour NO_2_ exposure, incorporating both same-hour exposures and complete diurnal patterns using a time-series design. The lag-specific effect was also computed, aiming to provide the risk associated with each hour. In the second stage, stratified analysis was performed for different patient categories based on sex, deprivation indices and ethnicity using a similar model design.

## Materials and method

Asthma emergency department admissions data from four acute hospitals managed by the University Hospitals Birmingham NHS Foundation Trust (UHB-NHSFT) were obtained from the PIONEER Health Data Research Hub for Acute Care in the West Midlands.^19^ For exposure, the study focuses on high-temporal-resolution hourly NO_2_ concentration.

### Study design and setting

This study used a time-series design to analyse hourly emergency hospital admissions for adult patients with acute asthma during the period from 1 June 2016 to 31 May 2022. It examined the association between temporal changes in NO_2_ concentrations over 0–24 hours and hourly emergency hospital admissions for adult patients with acute asthma living in the Birmingham and Solihull local authority areas within the West Midlands, UK. Birmingham and Solihull are among the most densely populated areas of the region (total population ~1.3 million), with an estimated 345 annual incidents of morbidity and mortality of adult asthma attributable to air pollution.^17^ The Birmingham and Solihull Integrated Care System has reported some of the highest hospital admission and mortality rates for lung conditions in England (https://fingertips.phe.org.uk/).

### Study population

**Figure 1:**
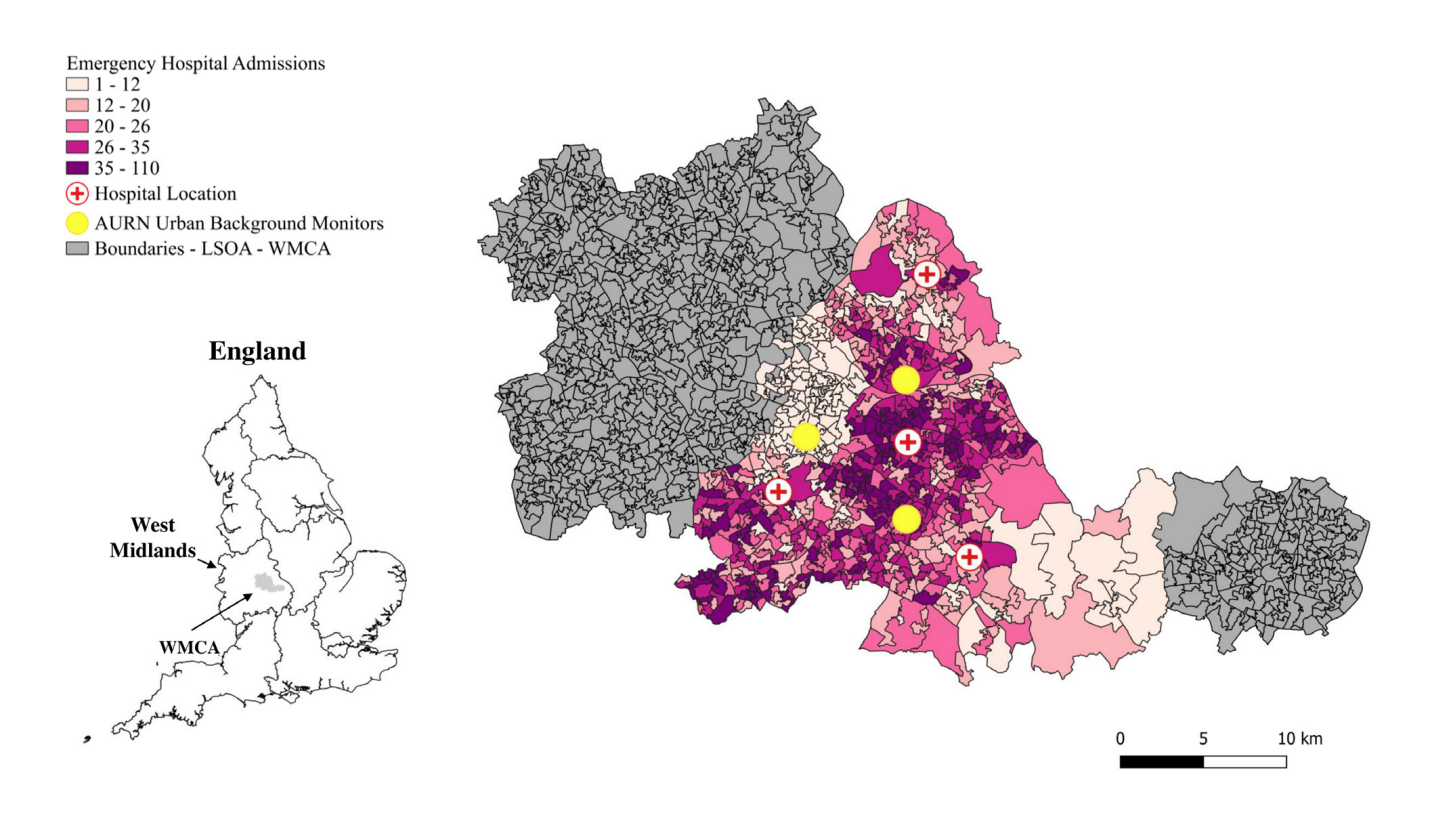
This map shows emergency hospital admissions across Lower Super Output Areas (LSOAs) in the Birmingham city and Solihull local authority postcode areas within the West Midlands Combined Authority (WMCA) region from 1 June 2016 to 31 May 2022. Darker purple shades indicate higher hospital admission frequencies. Red crosses mark the four hospital sites, which are managed by the University Hospitals Birmingham NHS Foundation Trust (UHB-NHSFT). Yellow circles indicate the locations of the Department for Environment, Food and Rural Affairs (Defra) Automatic Urban and Rural Network (AURN) urban background monitors. The small map of England highlights the location of WMCA in the West Midlands, UK.

Electronic health records (EHRs) of hospital admissions for acute asthma were retrieved from four acute hospitals in Birmingham and Solihull managed by UHB-NHSFT, which is among the largest national hospital services in the UK. It provides acute services at four hospitals: Good Hope Hospital, Heartlands Hospital, Queen Elizabeth Hospital Birmingham, and Solihull Hospital, serving a combined population of 1.3 million residents. The locations of these hospital sites are shown in figure 1.

### Data collection

#### Health outcome data

The outcome was the number of hourly emergency hospital admissions for acute asthma. Emergency hospital admission data for acute asthma were obtained from the PIONEER Health Data Research Hub for Acute Care, led by the UHB-NHSFT, UK.^20^ The data included hospital arrival date and hour for each patient with acute asthma, resulting in high-temporal-resolution data that are crucial for assessing the impact of extreme environmental changes. The cases were extracted based on the inclusion criteria of age ≥16 years at the time of admission. Further, it includes patients admitted with International Classification of Diseases diagnosis codes J45 or J46 (primary or secondary). All cases with confirmed COVID-19 based on laboratory testing (U07.1) and inconclusive COVID-19 cases (U07.2; virus not identified), that is, diagnosed clinically or epidemiologically but with inconclusive or unavailable laboratory testing based on either the primary or secondary diagnostics, were excluded from the analysis. Furthermore, for Solihull Hospital, the study period was different due to the closure of the Solihull emergency department post-April 2020. Therefore, the study period for patients from Solihull was from 1 June 2016 to 30 April 2020. Birmingham City and Solihull, as the primary catchment areas for four acute-care hospitals, account for the majority of emergency admissions in the dataset. After applying all inclusion and exclusion criteria, the combined total was 18 943 cases. In contrast, other West Midlands Combined Authority (WMCA) districts reported fewer than 300 admissions each, as shown in online supplemental figure S1. Therefore, these districts were excluded from the analysis, and the study was restricted to patients resident in Birmingham City and Solihull to avoid exposure misclassification. Figure 1 shows the spatial distribution of the number of hospital emergency admissions across Lower Super Output Areas in Birmingham City and Solihull local authority postcode areas within the WMCA region. Additionally, the EHR included information such as age, sex, socioeconomic status, and ethnicity of patients. The socioeconomic status of the area of residence was determined using English Indices of Deprivation scores obtained using postcodes.^21 22^ Ethnicity was self-reported during hospital arrival, and categories of these ethnic groups are provided in online supplemental table S1. Ethnicity classification followed national guidelines.^23^ In the stratified analysis, hourly hospital admissions for acute asthma were assessed by sex, deprivation indices, and ethnicity. Although classification followed the criteria mentioned, only broader categories were included in the risk assessment due to the smaller sample size. For instance, ethnicity was categorised as White and non-White, while the association with deprivation was assessed only for patients living in the most deprived and least deprived areas (quintiles 1 and 5). The majority of patients were of White ethnic background (including White British, White Irish and other White groups), of whom 97% were White British. Non-White patients included all other ethnic categories, including mixed ethnicities. The patients under the ‘not-known’ ethnicity category were excluded from the analysis.

#### Exposure data

The environmental data, such as hourly NO_2_, were obtained from urban background monitors in the Defra-managed Automatic Urban and Rural Network (AURN), which provides an integrated contribution from all sources upwind of the station. The study includes three urban background monitors located in Birmingham (UKA00559, UKA00655 and UKA00479), as shown in figure 1. The data from these monitors were downloaded from the publicly available Defra platform.^24^ As Solihull does not have an urban background monitor, monitoring records from Birmingham were used to estimate exposure for patients residing in the Solihull postcode areas. The data for other pollutants and meteorological parameters, including PM_2.5_ and wind speed, were also obtained from Defra. This study employs a time-series analysis to assess the relationship between hourly NO_2_ exposures and emergency hospital admissions for acute asthma in adults. The analysis primarily focused on admissions occurring between 08:00 and 21:00, referred to as daytime admissions. Night-time characteristics differ in terms of admission patterns, such as asthma severity, patient behaviour, sleep patterns, and more difficult access to healthcare, causing admission patterns during the day and night to be fundamentally different.^25^ However, for daytime visits, we considered overall 24-hour exposure, including daytime and night-time exposures. For example, for someone admitted at 9:00, their exposure at 7:00 is very relevant, despite this being during the night. By evaluating 24-hour exposure in relation to daytime admissions, the analysis effectively accounts for potential lagged effects over the full 24-hour period.

### Statistical analysis

A time-series Poisson generalised additive model, combined with a distributed lag non-linear model (DLNM), was used to estimate the short-term association between hourly NO_2_ exposure and emergency hospital admissions for acute asthma in patients aged 16 years and older. The main model fitted is given as follows:

log(*E*(*Y*_*t*_))=*α*+cb(NO_2_)+cb(temp)+ns(time, 7×year)+factor(dow)+bs(hour, df=11)+ns(ws)+ns(PM_2.5_)+SI

*Y*_*t*_ was the hourly count of hospital admissions for adult patients with acute asthma. A cross-basis (cb) function with basis (B)-splines (bs) for both hourly NO_2_ levels and time lag was used in the model to account for non-linearity and the delayed effect. The basis functions include no internal knots, use degree 3 and have boundary knots selected based on the minimum Akaike Information Criterion (AIC). Although the AIC was slightly lower for the linear spline than for the B-spline in the cross-basis of NO_2_, B-splines were chosen to better capture the complex nature of hourly variation, as shown in online supplemental table S2. The lag was modelled for 0–24 hours to account for complete 24-hour temporal variability. Temperature is a potential confounder for air pollution and respiratory diseases.^26 27^ The model was adjusted using a cross-basis function for hourly mean temperature and a 24-hour lag. In the temperature dimension, B-splines with equal knots were used, with 3 degrees of freedom (df). For the lag dimension, B-splines with logarithmically spaced knots were applied, using 4 df. The choice of these splines and df was based on the minimum AIC, as provided in online supplemental table S3. Time was controlled in the model using a smooth function of time with 7 df per year. These smooth functions control for seasonality and between-year patterns. Furthermore, the day of the week (dow) and the hour of the day were important confounders to control for weekly and hourly variations in NO_2_ due to different traffic patterns. The dow affects admissions because people behave differently on different days. Controlling for these variables ensures that observed associations are not merely artefacts of routine weekly or hourly patterns, but instead reflect actual relationships between NO_2_ concentration and hourly hospital admissions. Therefore, including the hour of the day ensures that NO_2_ variation above or below typical levels for a given hour is associated with increased asthma admissions. The hour of the day was controlled as B-splines with 11 df, obtained based on the lowest AIC (online supplemental table S4).

The model also included natural splines (ns) for PM_2.5_ and wind speed (ws) with no internal knots. The model choices were tested for sensitivity by fitting the model to different specifications, and selections were based on the lowest AIC values (online supplemental table S5). In addition, although the study only included the code for asthma, it also included the COVID-19 period; therefore, the model was controlled for the Stringency Index (SI). The SI was obtained from the Oxford Coronavirus Government Response Tracker project^28^ and represents a composite measure of nine government response indicators, including school closures, workplace closures, cancellation of public events, restrictions on public gatherings, closures of public transport, stay-at-home requirements, public information campaigns, restrictions on internal movements, and international travel controls. The index ranges from 0 to 100, with higher values indicating stricter governmental responses. The index represents the restrictions imposed during the COVID-19 period, which might cause fewer hospital admissions and lower exposure.^29^ Further, a segmented assessment was performed for the pre-COVID period (2016–2019). The relative risk (RR) was computed using 0 µg/m³ of NO_2_ (no exposure) as the reference, which serves as a counterfactual condition for comparison. The risk of hospital admissions using the 25th percentile of NO_2_ (7.3 µg/m³) as a reference (rather than zero) was also explored. In the second stage, stratified analyses were conducted for specific participant groups using the same model specifications, with a few exceptions, such as modifications to the time spline for seasonal analysis and for Solihull Hospital due to different temporal recordings. As the analyses were primarily subgroup-specific and stratified by participant characteristics, formal interaction analyses were not performed. All statistical analyses were conducted in R software, and the modelling was performed using the dlnm package.^30^

### Patient and public involvement

Patients or the public were not involved in the design, conduct, reporting or dissemination plans of this study.

## Results

There were a total of 18 943 emergency hospital admissions of adults with a diagnosis of acute asthma during the study period from 1 June 2016 to 31 May 2022. The majority of patients had residential addresses within the Birmingham city postcode area (n=15 908), while 3035 had addresses within the Solihull postcode area. The final analysis included 18 933 cases due to missing arrival time information (n=10). The majority of emergency hospital admissions were observed during the daytime (08:00–21:00), accounting for 78% of total hospital admissions, while the remaining cases were during night-time. Emergency hospital admissions were comparable between the cold (October–March) and warm (April–September) seasons. During the study period, of the total number of hospital admissions, 10 157 (54%) occurred during the cold months, while 8776 (46%) occurred in warm months. Characteristics of participants, including demographic and socioeconomic information, are presented in table 1. Among all adult emergency admissions, approximately 65% (n=12 268) were female. Additionally, a higher proportion of the population was classified as living in the most deprived areas (quintile 1) (~60%), while only 7% were categorised as living in the least deprived areas (quintile 5).

**Table 1:** Characteristics of emergency hospital admissions for adult (aged ≥16 years) patients with acute asthma from 1 June 2016 to 31 May 2022 in the Birmingham–Solihull metropolitan area, West Midlands, UK

Characteristics	Count of emergency hospitalisation	Percentage of emergency hospitalisation
Hospital		
Queen Elizabeth Hospital	6814	36
Birmingham Heartlands Hospital	6930	37
Good Hope Hospital	2969	16
Solihull Hospital	2230	12
Gender		
Female	12 268	65
Male	6675	35
Age (years)		
16–64	14 868	78
65+	4075	22
IMD		
1 (most deprived)	11 349	60
2	2953	16
3	2173	11
4	1176	6
5 (least deprived)	1292	7
Ethnicity		
White	10 583	56
Non-White/mixed	5664	30
Not known	2696	14
Season		
Summer (April–September)	8776	46
Winter (October–March)	10 157	54
Time		
Daytime (08:00–21:00)	14 796	78
Night-time (21:00–08:00)	4137	22

IMD, Index of Multiple Deprivation.

**Figure 2:**
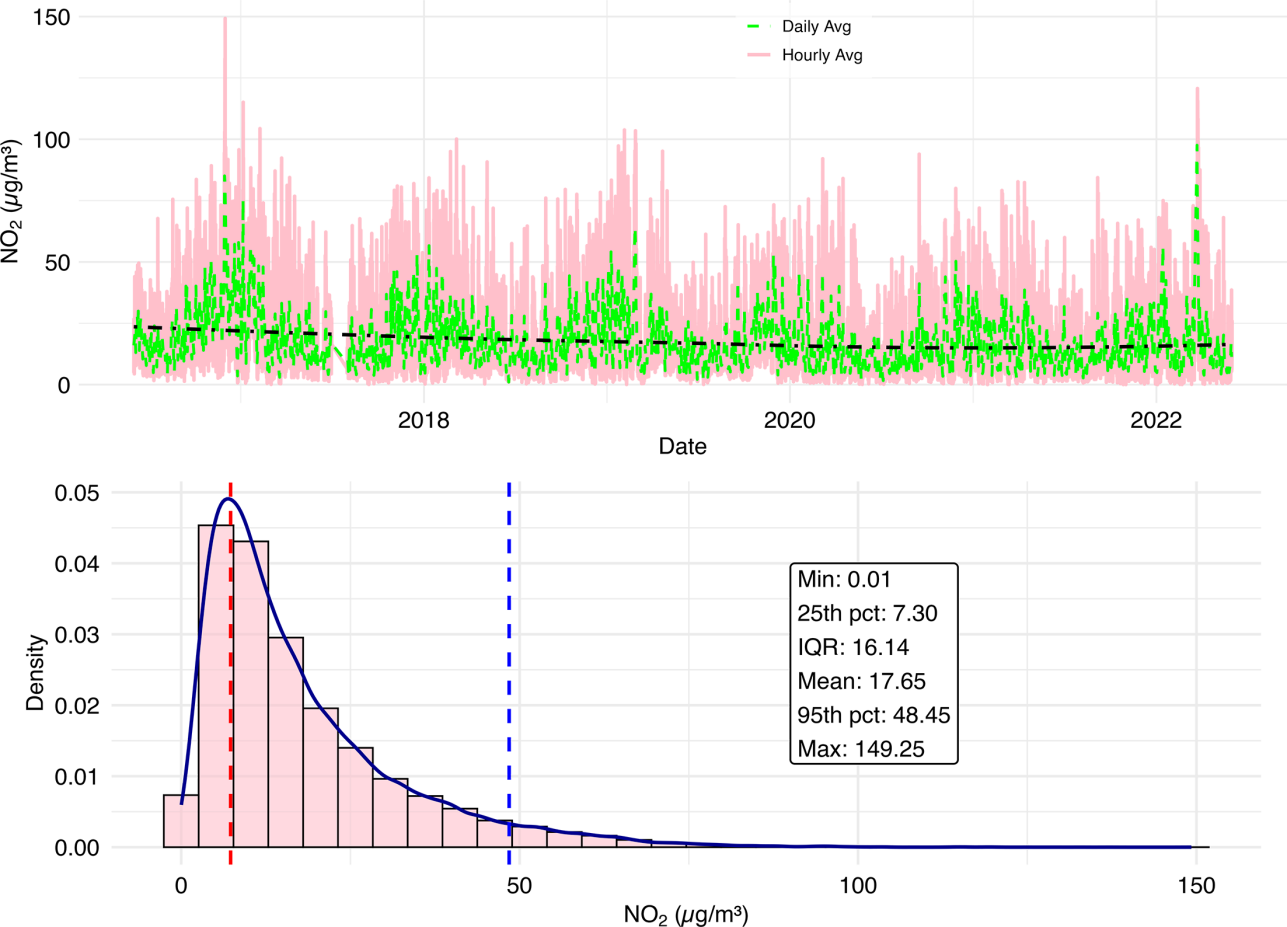
Hourly and daily averages of nitrogen dioxide (NO₂) concentrations (µg/m³) (top) and distribution of hourly NO_2_ (bottom) for Birmingham, West Midlands, UK, from 1 June 2016 to 31 May 2022. The black dashed line in the top plot represents the trend in NO_2_ concentration during the study period. The bottom plot includes the distribution of hourly NO_2_ and the minimum (min), 25th percentile (pct), IQR, mean, 95th percentile (pct) and maximum (max) values of NO_2_. In the bottom plot, the vertical red and blue dashed lines represent the 25th and 95th NO_2_ percentiles, respectively.

**Figure 3:**
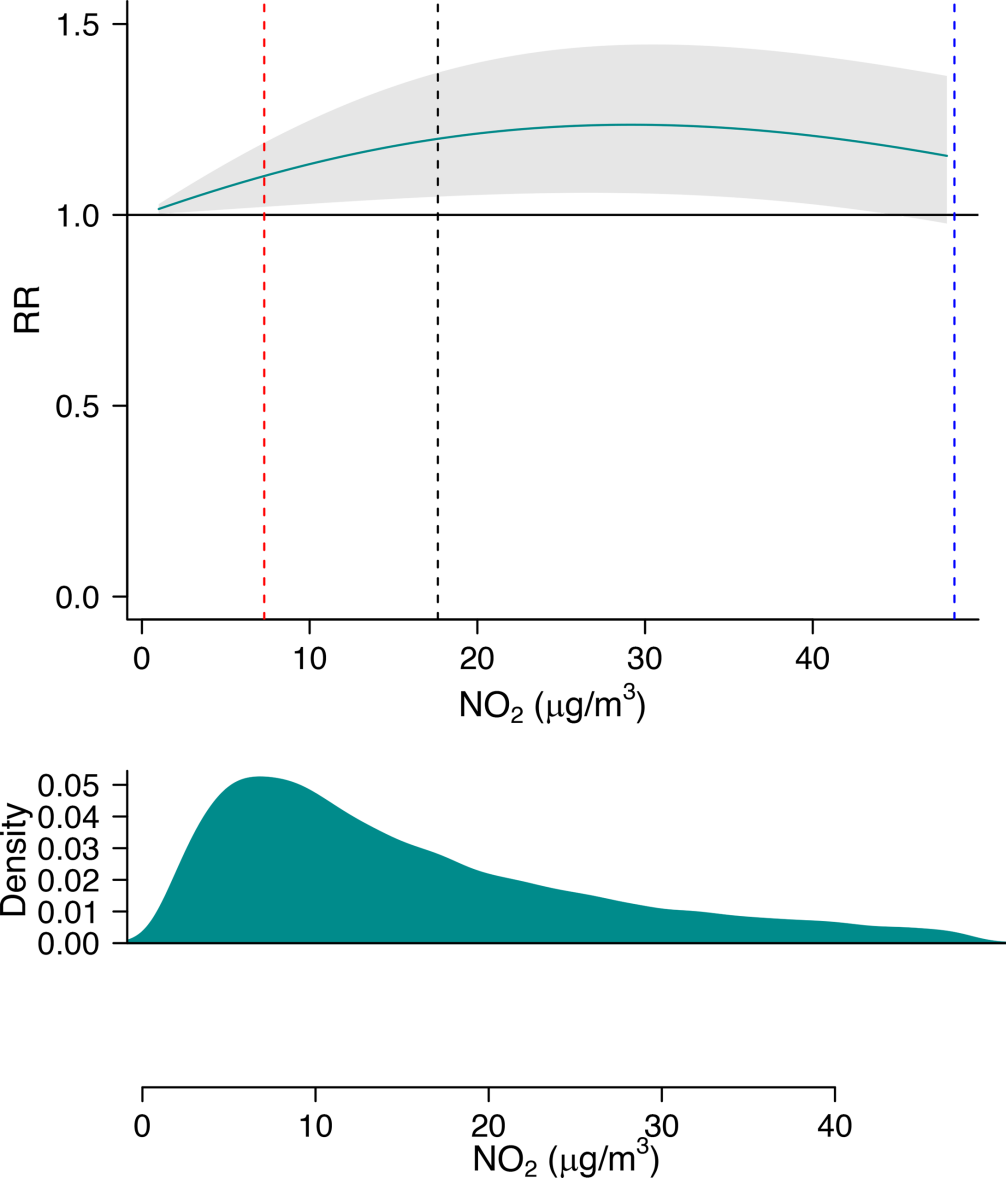
The cumulative exposure–response curves for the associations between nitrogen dioxide (NO_2_) and daytime emergency hospital admissions among adult patients with asthma over a lag of 0–24 hours using a reference of 0 µg/m^3^ (top) and the distribution of NO_2_ up to the 95th percentile during the study period (bottom). Note: the green line shows the cumulative relative risk (RR) of emergency hospital admissions for asthma, and the grey areas are the 95% CIs. The vertical dashed black lines represent the mean NO_2_ and vertical dashed red and blue represent the 25th percentile and 95th percentile of NO_2_.

Subsequently, figure 2 presents the hourly concentration of NO_2_ as well as the aggregated daily level from the urban background AURN monitoring locations in Birmingham, UK, from 1 June 2016 to 31 May 2022. Additionally, it shows the distribution of hourly NO_2_ for the study period. The mean NO_2_ concentrations during the daytime (08:00–21:00) and night-time (21:00–08:00) were observed to be 18 µg/m³ and 17 µg/m³, respectively. The 95th percentile of NO_2_ during the study period for daytime and night-time was 49 µg/m³ and 47 µg/m³, respectively. The maximum hourly concentration of NO_2_ was 149 µg/m³, recorded on 30 November 2016, at 08:00. The hourly distribution of pollutants reveals some extreme values for NO_2_. Furthermore, a decline in NO_2_ concentrations has been observed from 2016 to 2022, as depicted by the trend line in figure 3, with the highest annual mean concentration recorded in 2016 (24.4 μg/m³) and the lowest in 2020 (14.3 μg/m³), which may be attributed to the COVID-19 pandemic. However, the lower levels observed during 2021 and 2022 could also be attributed to NO_2_-focused interventions implemented through the Clean Air Zone (CAZ), which resulted in approximately 3.4% and 5.4% reductions in roadside NO_2_ concentrations in Birmingham, UK.^31^Online supplemental figure S4 illustrates hourly concentrations of NO_2_ across the study areas during the study period. It shows the high temporal variability of NO_2_ over the 24-hour cycle, with elevated values during morning and evening hours. The online supplemental figure 4 also presents seasonal and weekly variability, with higher concentrations during weekdays and lower levels during weekends. Furthermore, the online supplemental figure 4 highlights reduced NO_2_ levels during the COVID-19 period and post-COVID compared with the pre-COVID period.

### Association of NO_2_ and emergency hospital admissions for acute asthma

The study showed a positive association between NO_2_ levels and daytime emergency hospital admissions among adult patients with acute asthma over a 24-hour lag. The overall cumulative RR for mean NO_2_ (18 µg/m³), using a controlled model and a reference of zero exposure, was 1.13 (95% CI 1.01 to 1.26). In addition, for every 10 µg/m³ increase in NO_2_ levels, the overall cumulative risk of emergency hospital admissions was observed to be 1.11 (95% CI 1.01 to 1.23). Figure 3 illustrates the cumulative exposure–response curves for the associations between NO_2_ and the daytime emergency hospital admissions for acute asthma over a 0–24-hour lag. Figure 3 also includes the distribution of NO_2,_ which is moderately right-skewed, with most values around 5–15 (μg/m³) and fewer instances of higher concentrations of 48 µg/m³ (95th NO_2_ percentile). The cumulative RR for the 95th percentile of NO_2_ was 1.14 (95% CI 0.97 to 1.35), and estimates beyond the 95th percentile were not included in figure 3. The risk observed beyond the 95th percentile of NO_2_ was not considered a true association due to a wider CI; for instance, the risk for the 99th percentile NO_2_ (68 µg/m³) was 0.88 (95% CI 0.71 to 1.09). While the study primarily focused on assessing the daytime risk of admissions, for night-time, the overall cumulative RR for mean NO_2_ over a 24-hour lag, using 0 µg/m^3^ (no exposure) as the reference, was 1.02 (95% CI 0.84 to 1.29). In addition, for total day and night admissions, the overall cumulative RR was 1.12 (95% CI 1.02 to 1.22).

We also estimated the risk using the reference corresponding to the 25th percentile of NO_2_ (7.3 µg/m³). The cumulative risk for mean NO_2_ with reference to the 25th percentile NO_2_ was found to be 1.04 (95% CI 0.98 to 1.10). Additionally, considering that the study period included both COVID-19 restriction periods and the post-COVID period, which may have resulted in reduced hospital admissions and exposure, a segmented analysis was performed for the pre-COVID period (2016–2019). During this period, the overall cumulative RR for mean NO_2_ over the 24-hour lag, using a reference of zero exposure, was 1.06 (95% CI 0.92 to 1.23). The association during the pre-COVID period was observed to be statistically non-significant, which could be attributed to the smaller sample size. The model sensitivity was assessed using varying df for time and lag, with the choices based on AIC criteria. The results for the sensitivity analysis are included in the supplementary information (online supplemental table S6). The results for the sensitivity analysis comparing the Poisson and quasi-Poisson distributions are provided in the online supplemental figure S5. Furthermore, considering the importance of covariates such as PM_2.5_ and the hour of the day, the RRs comparing the output with the fully adjusted model and models without PM_2.5_ and the hour of the day are also provided in online supplemental figure S6.

**Figure 4:**
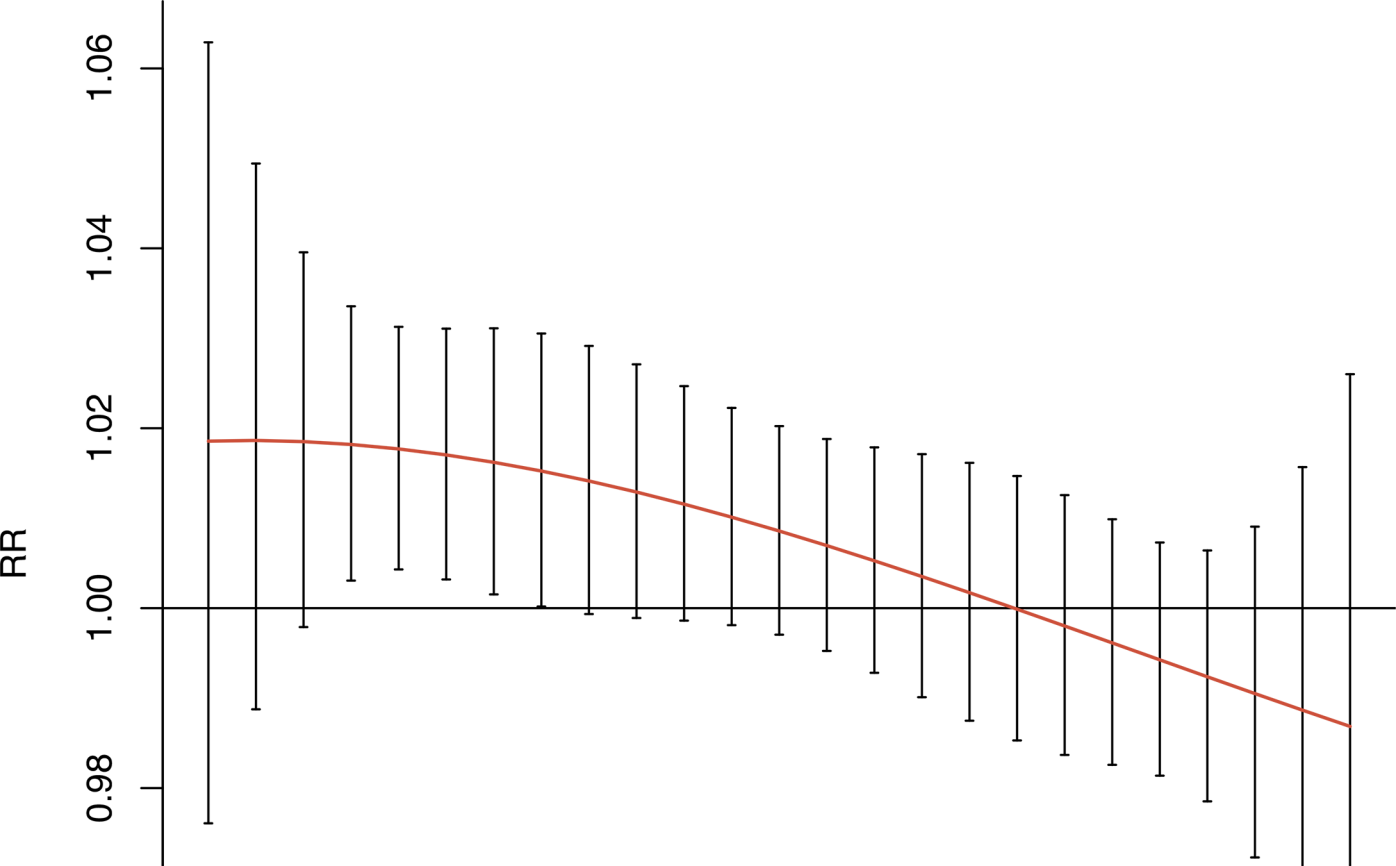
The relative risk (RR) of emergency hospital admissions associated with mean nitrogen dioxide at the specific lags from 0 to 24 hours. Note: the red line shows the average RR of emergency hospital admissions for acute asthma, and the error bars are the 95% CIs.

**Figure 5:**
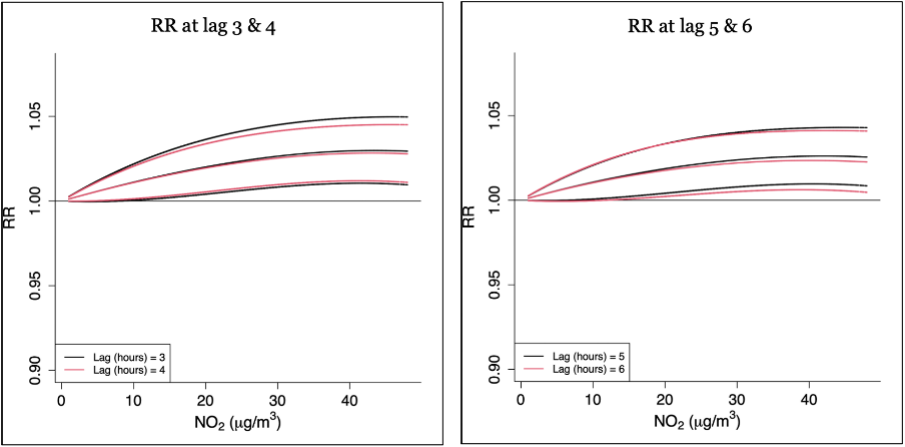
The exposure–response curves for the associations between nitrogen dioxide (NO_2_) and emergency hospital admissions for acute asthma at specific lags (left plot: lags 3 and 4 hours; right plot: lags 5 and 6 hours). Note: the solid red and black lines show the average relative risk (RR) of emergency hospital admissions for acute asthma, and the dashed lines are the 95% CIs for the respective lags.

The study also examined the risk of emergency hospital admissions due to mean NO_2_ exposure across specific lags from 0 to 24 hours, as shown in figure 4. The lag-specific analysis revealed a consistent and positive association at lags between 3 and 6 hours, while the effect declined at longer lags. The RR for lag 3 and lag 6 was 1.02 (95% CI 1.003 to 1.033) and 1.02 (95% CI 1.001 to 1.031), respectively. In addition, the exposure–response curves for the associations between NO_2_ and the risk of emergency hospital admission for acute asthma at these specific lags (3–6) were also observed as depicted in figure 5. Figures 4 and 5 also show consistent positive associations between NO_2_ and daytime hospital admissions for these hourly lags. This highlights the variability in the exposure–response relationship within 24 hours, emphasising the importance of using high-temporal-resolution exposure data to accurately capture the associated risks.

In the second stage, the cumulative RR with respect to zero exposure over a 24-hour lag was computed for emergency hospital admissions among stratified participants. These subgroup-specific estimates reflect the effect modification of the NO_2_-asthma association in specific participant groups, although no formal interaction term was tested within groups. Table 2 presents the participant-specific overall RR and their 95% CI for daytime emergency hospital admissions for mean NO_2_ levels during the study period in the Birmingham–Solihull metropolitan area, West Midlands, UK. The study primarily focused on hospital admissions to four hospitals, as listed in table 1. However, to understand the hospital-specific pattern between the two districts, the risk associated with each hospital was analysed individually for Queen Elizabeth Hospital in Birmingham and Solihull Hospital in Solihull. For Queen Elizabeth Hospital, the overall cumulative RR was 1.18 (95% CI 0.95 to 1.48). In contrast, the risk was lower for Solihull Hospital, where the study period was different due to the closure of the Solihull emergency department post-April 2020, resulting in fewer hospital admissions. Furthermore, the use of Birmingham’s urban background monitors for Solihull may introduce exposure misclassification, potentially underestimating risk in this subgroup. Additionally, the study assessed the association between emergency hospital admissions and risk to specific socioeconomic and demographic participants, including gender, deprivation indices, and ethnic background. In gender-specific associations, both males and females exhibited an increased risk of hospitalisation with exposure to hourly NO_2_. The RR for females was 1.14 (95% CI 0.97 to 1.34), while for males, the RR was 1.24 (95% CI 0.99 to 1.56). The association in patients with specific deprivation indices indicated a higher positive risk for patients living in the most deprived areas, with a risk estimate of 1.20 (95% CI 1.01 to 1.42) in quintile 1, compared with patients in the least deprived areas, who had a risk estimate of 1.05 (95% CI 0.63 to 1.74) in quintile 5. In ethnicity-specific analysis, non-White/mixed individuals had higher RRs than White individuals, although neither estimate reached statistical significance (table 2). The findings underscore the potential for increased risk among non-White/mixed individuals, although further refinement of ethnicity-specific groups, such as distinguishing Black and Asian populations within the non-White category, may provide more precise results. In addition, the seasonal effect was also computed with data stratified by specific months. The effect modification was notable for both warm and cold seasons, although neither estimate reached statistical significance. The participant-specific cumulative exposure–response curves and lag-specific risk plot are provided in the online supplemental figures 7 and 8.

**Table 2:** The overall daytime RR and its 95% CI for emergency hospital admissions for acute asthma in adult patients associated with the mean NO_2_ levels from June 2016 to May 2022 in the Birmingham–Solihull, West Midlands, UK

Scenario	RR	95% CI (low)	95% CI (high)
All daytime patients	1.13	1.01	1.26
Single hospital			
Queen Elizabeth Hospital	1.18	0.95	1.48
Solihull Hospital*	1.15	0.77	1.72
Gender			
Female	1.14	0.97	1.34
Male	1.24	0.99	1.56
IMD quintile*			
1 (most deprived area)	1.20	1.01	1.42
5 (least deprived area)	1.05	0.63	1.74
Ethnicity*			
White	1.14	0.95	1.36
Non-White/mixed	1.27	0.99	1.63
Seasons			
Summer season (April–September)	1.17	0.98	1.44
Winter season (November–March)	1.06	0.90	1.26

IMD, Index of Multiple Deprivation; NO_2_, nitrogen dioxide; RR, relative risk.

## Discussion

This time-series study evaluated the association between hourly ambient NO_2_ exposure and risk of emergency hospital admissions for patients with acute asthma aged 16 years and older living in residential postcodes within the Birmingham and Solihull local authority areas in the West Midlands. The study estimated the RR of hospital admissions due to NO_2_ exposure for a cumulative lag of 0–24 hours prior to the hour of arrival. The findings suggested a positive association between increasing hourly NO_2_ exposure and emergency hospital admissions for acute asthma exacerbations in adults. Furthermore, subgroup analysis indicated a significant positive association between hourly NO_2_ exposure and the risk of hospital admissions among patients living in the most deprived areas. Hourly data showed substantially high NO_2_ levels, with concentrations exceeding 100 µg/m³ in 0.05% of observations. The maximum hourly concentration recorded during the study period was 149 µg/m³. Although these episodes account for only 0.05% of the total data, exposure to such high levels and diurnal changes could significantly harm vulnerable populations.

Most previous studies have examined daily or monthly average exposures, thereby potentially missing short-term changes. For instance, a study reported a 4% increase in adult asthma admissions per 1 µg/m³ increase in mean monthly NO_2_ levels.^15^ Similarly, one-third of general practice and half of accident and emergency respiratory visits were attributed to daily mean NO_2_ levels exceeding the WHO 2021 global 24-hour air quality guideline threshold of 25 µg/m^3^.^32^ However, studies exploring the hourly association between NO_2_ and health outcomes remain limited. Notably, a study found that NO_2_ exposure with a 10-hour lag was associated with increased all-cause morbidity-related ambulance calls.^33^ Furthermore, studies have used time-stratified case-crossover designs to assess the association between hourly air pollution levels and asthma. For example, a time-stratified case-crossover study demonstrated a significant increase in asthma-related emergency department visits within the hour of exposure (lag 0 hour).^14^ The study reported an 18.1% (95% CI 15.4% to 21.0%) increased risk of asthma-related emergency department visits per IQR increase in NO_2_ levels (30 µg/m^3^) over 0–72 hours in a Chinese city. Additionally, it was found that short-term exposure to NO_2_ significantly influenced respiratory disease onset within 6–12 hours.^34^ However, the time-stratified case-crossover uses fixed time strata, which may fail to fully capture the temporal exposure–response pattern.^35^

Furthermore, in addition to assessing the overall cumulative risk for the 0–24-hour lag, this study also evaluated the risk for specific lags. While the same-hour exposure risk was substantial, it had a wider CI and included 1.00. However, a statistically significant and more confident risk was observed for a 3–6-hour lag, suggesting that 3 hours of NO_2_ exposure could be associated with emergency hospital admissions for acute asthma. A systematic review and meta-analysis found that 1-hour NO_2_ exposure did not show a significant association with asthma (RR 0.99 (95% CI 0.97 to 1.03) for maximum daily 1-hour NO_2_ exposure).^36^ However, the analysis may not be able to accurately assess the association as it focused on the 1-hour maximum exposure rather than considering the overall hourly pattern.

Subsequently, the study also examined the risk of daytime hospital admissions due to hourly exposure to NO_2_ across different stratified participants. In the analysis, solely performed on Solihull Hospital, the risk of hospital admissions was considerably lower in comparison with Elizabeth Hospital. There are a number of possible explanations for these differences, including different age structures, settings and service provisions between the two areas. Additionally, a study reported that, among the total air pollution-attributable asthma-related annual incidents of morbidity and mortality in the WMCA area, 7% were observed in Solihull and 41% in Birmingham.^17^ However, the study period for Solihull differed, with no admissions post-April 2020, as well as possible exposure misclassification, which may have contributed to the lower risk.

The study also explored the role of individual socioeconomic status and ethnicity in air pollution-associated asthma risks. For deprivation indices, there was a significant positive association between NO_2_ exposure and the risk of hospital admissions among those living in the most deprived areas. However, for those living in the least deprived areas, the risk was not accurately assessed due to a smaller sample size. A few studies have also reported the lowest effect among the least deprived individuals.^37 38^ Additionally, a suggestive positive association between daytime admissions for both the White and non-White minority groups and hourly NO_2_ exposure was found. However, due to the small sample size, further classification among non-White groups, such as South Asian and Black, was not performed. Therefore, results might differ after including other categories separately among the non-White groups. For instance, a study found that South-East Asian patients were more likely to experience increased asthma hospitalisations due to air pollution exposure and other contributing factors than White patients.^39^ The study also explored the hourly risk of hospital admissions specific to cold and warm seasons. Similar to previous studies on seasonal effects on health responses,^40^ both seasons indicated a positive association with emergency hospital admissions for asthma due to NO_2_ exposures. Additionally, the effect modification was higher for the warm season in comparison with the cold season, which is inconsistent with the literature. The discrepancy may be attributed to the limited number of studies examining the hourly association of NO_2_ with hospital admissions. However, the effect modification for both periods was not statistically significant. These findings highlight the need for further research with larger sample sizes to better understand the hourly seasonal associations between acute hospital admissions and NO_2_ concentrations.

Although all participant-specific associations showed a positive effect, the wider CIs, including the protective effect, limit the ability to draw firm conclusions. The wider CI may be attributed to the small sample size. These findings underscore the need for future studies using high-resolution hourly data to assess the participant-specific impact of hourly NO_2_ exposure.

### Strengths and limitations

A key strength of this study is the use of high-temporal-resolution data, allowing for a more granular assessment of exposure–response relationships. The variability in the exposure–response relationship within 24 hours emphasises the importance of using high-temporal-resolution exposure data to accurately capture the associated risks. However, it is important to note some limitations as well. The current study used aggregated monitoring network data, which may introduce spatial misclassification bias, employing high-spatial-resolution data or personal exposure monitoring could give more accurate estimates. For instance, a study used high-spatial-resolution data of 100×100 m for cities in the UK and demonstrated a strong association between NO_2_ exposure and annual paediatric asthma cases.^41^ Thus, investigating the association using high-spatial-temporal-resolution data is crucial for providing more information to healthcare facilities and enhancing their response during extreme environmental conditions. Furthermore, the findings for specific participants are only suggestive rather than conclusive due to the wider CI and the inclusion of 1.00 in the RR. While the model adjusted for temperature, PM_2.5_ and seasonality, unmeasured factors (eg, indoor NO_2_ exposure) could influence the observed association.^42^ Further work with a larger sample size could provide more confident and conclusive results for different groups. It should also be acknowledged that reliance on routine administrative data introduces the potential for misclassification, particularly regarding the timing and occurrence of health events, which is an inherent limitation of the present analysis.^43^

Furthermore, given the complexity of admission patterns during night-time and the limited sample size available for such cases, this analysis primarily focused on daytime hospital admissions. The reasons for night-time asthma admissions are multifaceted, encompassing acute asthma severity, healthcare access and patient behaviour.^25^ However, exposures occurring during the evening may contribute to admissions within the subsequent 3–6 hours, potentially leading to late-night admissions that were not captured in the present analysis. Therefore, future research should undertake dedicated investigations to understand the complex patterns of night-time admissions and their association with hourly exposures.

Additionally, the analysis specifically excluded patients with confirmed and suspected SARS-CoV-2 infection using the primary and secondary diagnoses. During the COVID-19 pandemic period, clinicians were advised to distinguish asthma exacerbations from COVID-19, using symptoms like high fever and loss of taste or smell to guide triage.^44^ Patients without COVID-19 signs were directed to routine asthma care, while those with potential symptoms were assessed in separate designated areas and tested for COVID-19. Triage protocols prioritised in-person care for patients with severe asthma or acute exacerbations, while those with stable or mild-to-moderate disease were managed remotely through telemedicine.^45^ Furthermore, the routine face-to-face visits were deferred, with a focus on virtual consultations to maintain continuity of care, and patients were encouraged to follow their asthma action plans. It is clear that these changes will have affected the admission rates in our data, but we have accounted for them to the best of our ability by using the SI.

In addition, the restrictions during the COVID-19 period resulted in a notable reduction in NO_2_ concentrations, with a declining trend from January 2020 until the end of 2022 compared with previous years. The implementation of CAZ has further contributed to sustained reductions in NO_2_ levels.^31^ This underscores a significant improvement in air quality since 2020. Unlike changes to admissions, changes in exposure do not confound or bias our results; instead, they act as a natural experiment that increases the range of exposures observed. However, a direct association between the reduced NO_2_ levels and asthma hospitalisations was not assessed in this study due to the small sample size. Future work aims to assess the direct association between reduced NO_2_ levels and asthma hospitalisations to further strengthen these findings. Nonetheless, given the significant overall association observed between hourly NO_2_ levels and acute asthma admissions, the current results reinforce the importance of interventions targeting air quality improvement, such as CAZ and the adoption of cleaner fuels for transport and industrial activities.

There are various other predictors for asthma attacks, such as blood eosinophil count, fractional exhaled nitric oxide (FeNO), asthma attack history, disease severity, low Forced Expiratory Volume in 1 Second (FEV_1_%) and symptoms (Asthma Control Questionnaire (ACQ-5) score). A study reported that a 10% decrement in baseline FEV₁% predicted corresponds to an RR of 1.11 (95% CI 1.08 to 1.15) and a 0.5 increase in ACQ-5 score corresponds to an RR of 1.10 (95% CI 1.07 to 1.13), which is lower than the risk of exacerbation associated with NO_2_ as observed in this analysis.^46^ However, future studies should directly compare these predictors for asthma attacks with hourly exposures to traffic-related pollutants such as NO_2._

## Conclusions

This study strengthens the evidence for the importance of using high-temporal-resolution air pollution data to examine impacts on healthcare, particularly where there are marked temporal patterns in exposure magnitude. Results provide some evidence that hourly NO_2_ exposure is associated with an increased risk of emergency hospital admissions for acute asthma in adults, particularly for 3–6 hours of exposure prior to admissions. A higher percentage of emergency hospital admissions and higher statistical certainty were seen in those from the most deprived residential areas. Future work should focus on understanding short-term exposure–response relationships to provide evidence for NHS and public health preparedness, resource allocation, and pollution mitigation strategies.

## Supplementary material

10.1136/bmjopen-2025-110612 online supplemental figure 1

10.1136/bmjopen-2025-110612 online supplemental figure 2

10.1136/bmjopen-2025-110612 online supplemental figure 3

10.1136/bmjopen-2025-110612 online supplemental figure 4

10.1136/bmjopen-2025-110612 online supplemental figure 5

10.1136/bmjopen-2025-110612 online supplemental figure 6

10.1136/bmjopen-2025-110612 online supplemental figure 7

10.1136/bmjopen-2025-110612 online supplemental figure 8

10.1136/bmjopen-2025-110612 online supplemental table 1

## Data Availability

Data may be obtained from a third party and are not publicly available.
