# Supplementary material for: Does hourly variation in ambient nitrogen dioxide increase the risk of acute asthma hospitalisations? A time-series study of Birmingham and Solihull, UK
Source: BMJ Open. 2026 Aug 7;16(8):e110612. doi: 10.1136/bmjopen-2025-110612 (PMC13475568; doi:10.1136/bmjopen-2025-110612)
Supplement: Supplementary file 1 [file bmjopen-16-8-s002.pdf]

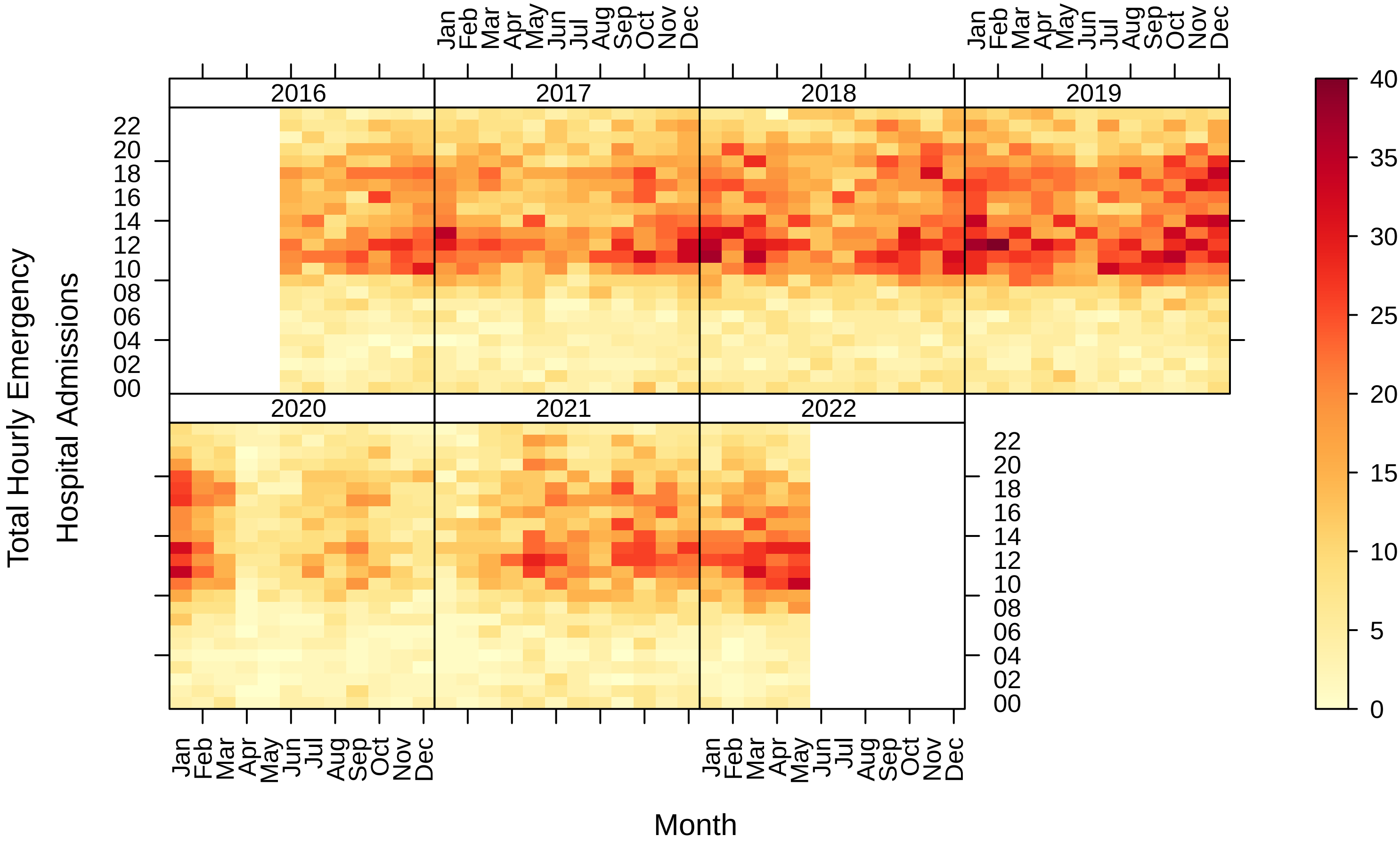

Total Hourly Emergency
Hospital Admissions
Month
2016
2017
2018
2019
2020
2021
2022
Jan
Feb
Mar
Apr
May
Jun
Jul
Aug
Sep
Oct
Nov
Dec
00
02
04
06
08
10
12
14
16
18
20
22
0
5
10
15
20
25
30
35
40
