# Supplementary material for: Does hourly variation in ambient nitrogen dioxide increase the risk of acute asthma hospitalisations? A time-series study of Birmingham and Solihull, UK
Source: BMJ Open. 2026 Aug 7;16(8):e110612. doi: 10.1136/bmjopen-2025-110612 (PMC13475568; doi:10.1136/bmjopen-2025-110612)
Supplement: Supplementary file 2 [file bmjopen-16-8-s003.pdf]

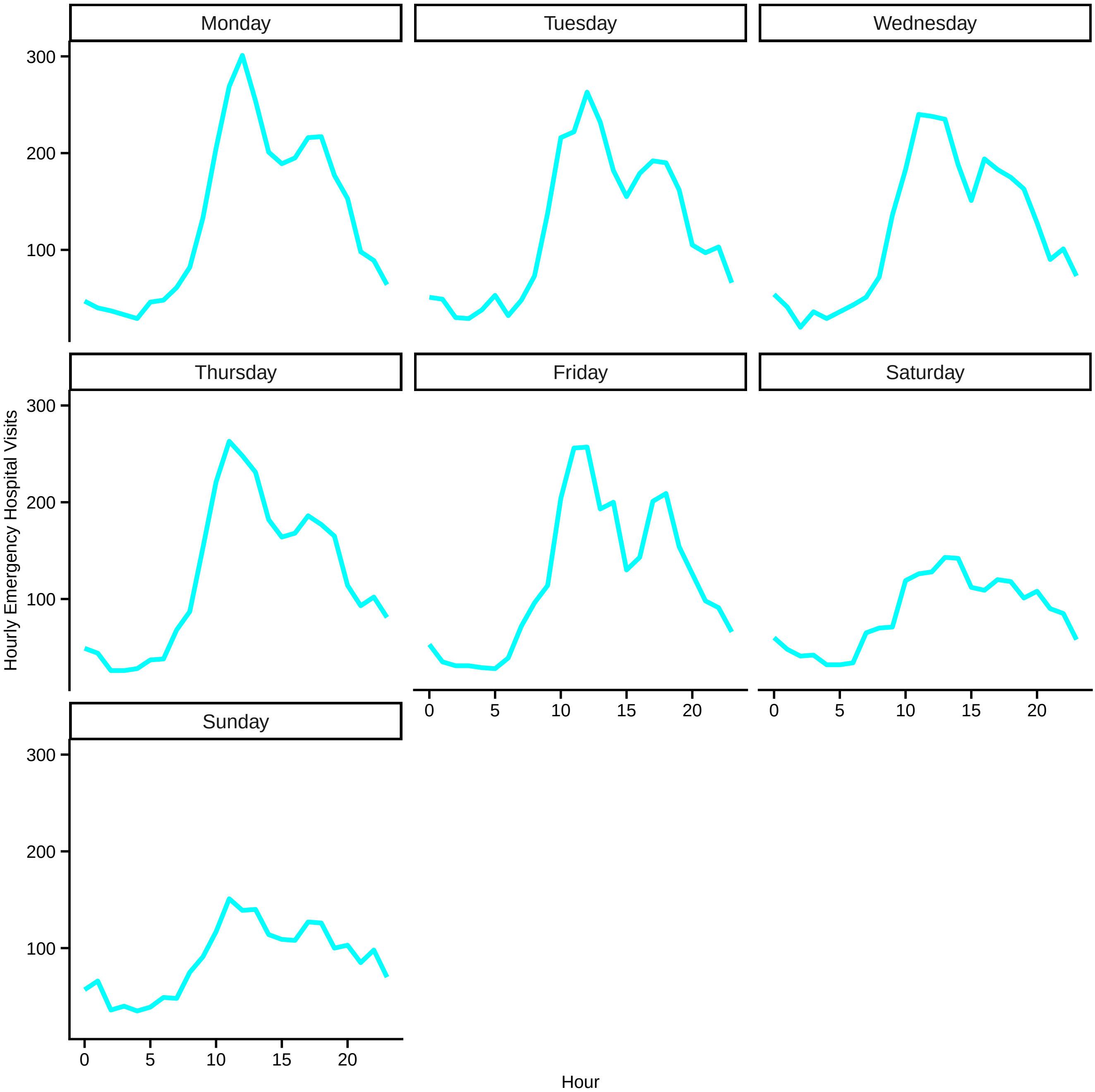
Monday
Tuesday
Wednesday
Thursday
Friday
Saturday
Sunday
300
200
100
0
5
10
15
20
Hourly Emergency Hospital Visits
Hour
