# Supplementary material for: Does hourly variation in ambient nitrogen dioxide increase the risk of acute asthma hospitalisations? A time-series study of Birmingham and Solihull, UK
Source: BMJ Open. 2026 Aug 7;16(8):e110612. doi: 10.1136/bmjopen-2025-110612 (PMC13475568; doi:10.1136/bmjopen-2025-110612)
Supplement: Supplementary file 4 [file bmjopen-16-8-s006.pdf]

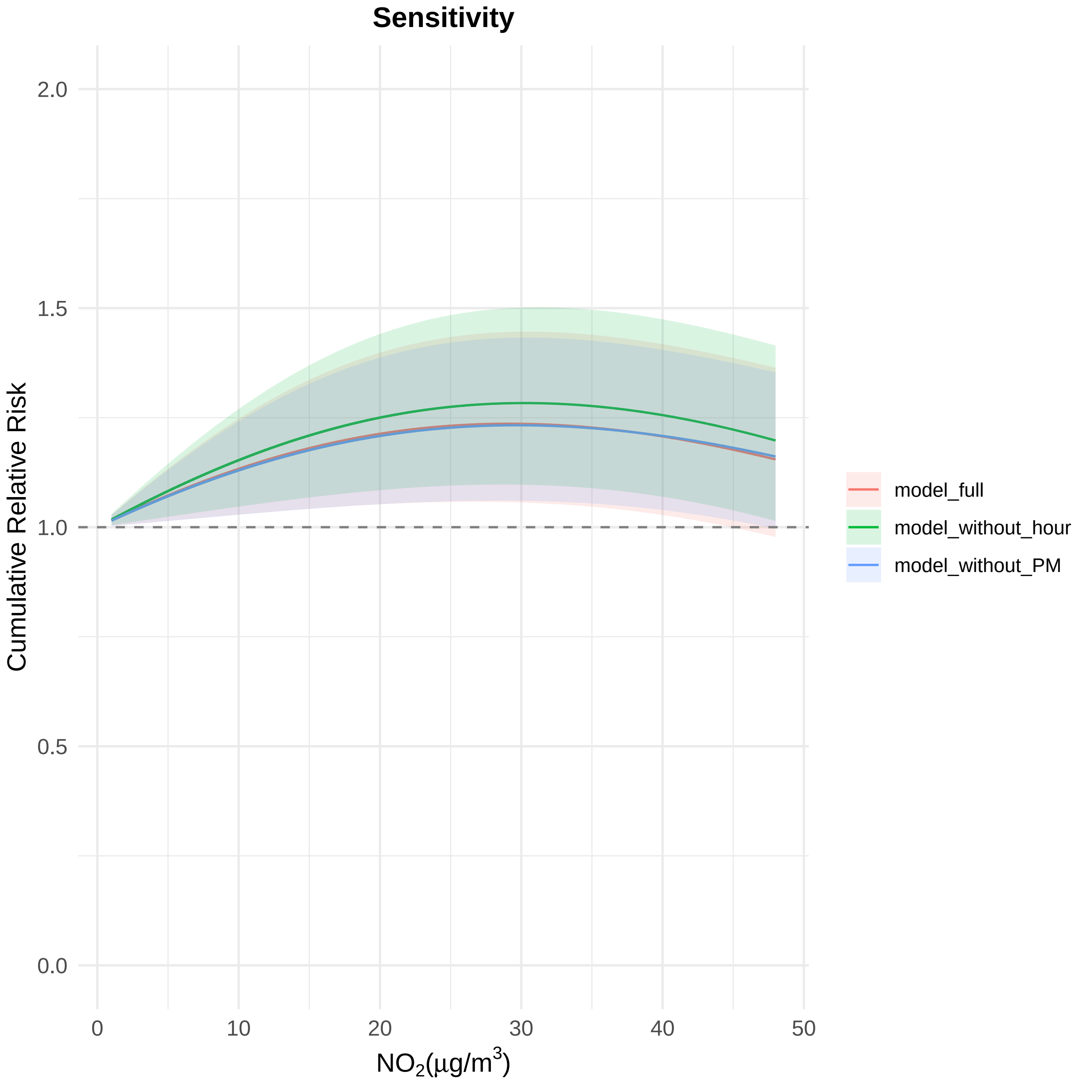
Sensitivity
Cumulative Relative Risk
2.0
1.5
1.0
0.5
0.0
0
10
20
30
40
50
NO2(μg/m3)
model_full
model_without_hour
model_without_PM
