# Supplementary material for: Does hourly variation in ambient nitrogen dioxide increase the risk of acute asthma hospitalisations? A time-series study of Birmingham and Solihull, UK
Source: BMJ Open. 2026 Aug 7;16(8):e110612. doi: 10.1136/bmjopen-2025-110612 (PMC13475568; doi:10.1136/bmjopen-2025-110612)
Supplement: Supplementary file 6 [file bmjopen-16-8-s008.pdf]

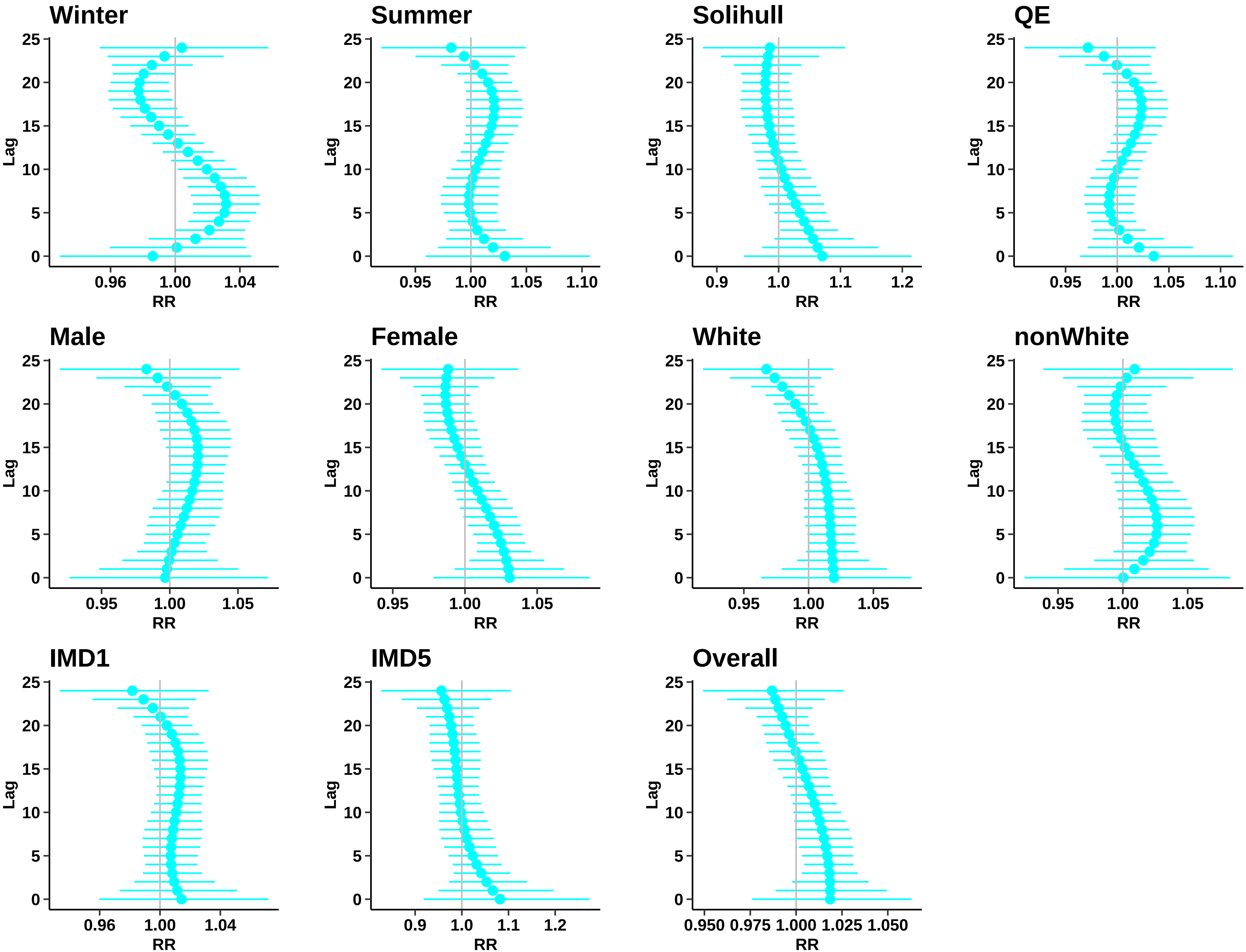
Winter
Summer
Solihull
QE
Male
Female
White
nonWhite
IMD1
IMD5
Overall
Lag
RR
0
5
10
15
20
25
0.96
1.00
1.04
0.95
1.05
1.10
0.9
1.0
1.1
1.2
0.950
0.975
1.000
1.025
1.050
