# Supplementary material for: Does hourly variation in ambient nitrogen dioxide increase the risk of acute asthma hospitalisations? A time-series study of Birmingham and Solihull, UK
Source: BMJ Open. 2026 Aug 7;16(8):e110612. doi: 10.1136/bmjopen-2025-110612 (PMC13475568; doi:10.1136/bmjopen-2025-110612)
Supplement: Supplementary file 7 [file bmjopen-16-8-s009.pdf]

Supplementary Information

Table S1: Categories of Ethnic groups in the dataset.

**Ethnic Group**
White - British
Not known
Asian/Asian Brit - Pakistani
Asian/Asian Brit - Indian
Any Other Ethnic Group Asian/Asian Brit-any oth Asian b/g
Black/Blk Brit-Caribbean
Black/Blk Brit-African
White - Irish
Asian/Asian Brit - Bangladeshi
White - any other White b/g
Black/Blk Brit-Any oth Blk b/g
Mixed-any oth mixed background
Mixed-White & Black Caribbean
Mixed-White & Asian
Mixed-White & Black African
Other Ethnic Group - Chinese
Other Ethnic Group

Table S2: Akaike Information Criteria (AIC) for selecting df and Knots for the cross-basis of NO_2.

Knot	AIC
1	**43024**
2	43024
3	43025
4	43025
DoF	AIC
1	**43026**
2	43026
3	43026
4	43032

Splines for Var	AIC

Lin	43032
**B-splines**	**43025**
P-splines	43056
Natural Splines	43032

Splines for Lag	AIC
Linear	**43020**
B-splines	**43026**
P-splines	43025
Natural Splines	43020

Table S3: Akaike Information Criteria (AIC) for selecting degrees of freedom (df) for cross-basis of temperature.

**df Var**	**df Lag**	**AIC**
**3**	**4**	**43026**
3	5	43028
3	6	43030
4	4	43030
4	5	43034
4	6	43036
5	4	43035
5	5	43039
5	6	43042
6	4	43039
6	5	43045
6	6	43048

Table S4. Akaike Information Criteria (AIC) for selecting degrees of freedom (df) and splines for "hour of the day" in the model.

**df**	**ns**	**bs**
0	43599	43205
1	43599	43205
2	43316	43205
3	43137	43205
4	43144	43146
5	43042	43045

6	43034	43081
7	43050	43044
8	43031	**43026**
9	**43026**	43050
10	43031	43031
11	43027	**43026**
12	43029	43031
13	43031	43027
14	43029	43029
15	43031	43031
16	43033	43029
17	43032	43030
18	43034	43033
19	43033	43032
20	43034	43034
21	43034	43033
22	43034	43034
23	43034	43034
24	43034	43034

Table S5. Akaike Information Criteria (AIC) for selecting degrees of freedom (df) $PM_{2.5}$ and Wind Speed.

Confounder	df	AIC
PM	1	43026
	2	43027
	3	43029
	4	43031
	5	43033
	6	43034
Wind Speed (ws)	1	43026
	2	43027
	3	43029
	4	43030
	5	43031
	6	43032

Table S6. Cumulative RR for the associations between NO_2 and daytime emergency hospital admissions of adult asthmatic patients for different Lag and time splines.

Variable	Specification	RR	RR Low	RR High
**Lag**	**Lag 4**	1.08	0.99	1.18
	**Lag 8**	1.08	0.99	1.19
	**Lag 12**	1.10	1.00	1.22
	**Lag 16**	1.14	1.03	1.26
	**Lag 24**	1.14	1.02	1.28
**Time Spline**	**1*7**	1.13	1.02	1.26
	**2*7**	1.10	0.99	1.23
	**4*7**	1.13	1.01	1.27
	**6*7**	1.14	1.02	1.28
	**7*7**	1.14	1.02	1.28
