# Supplementary material for: Does hourly variation in ambient nitrogen dioxide increase the risk of acute asthma hospitalisations? A time-series study of Birmingham and Solihull, UK
Source: BMJ Open. 2026 Aug 7;16(8):e110612. doi: 10.1136/bmjopen-2025-110612 (PMC13475568; doi:10.1136/bmjopen-2025-110612)
Supplement: Supplementary file 8 [file bmjopen-2025-110612.reviewer_comments.pdf]

PEER REVIEW HISTORY

BMJ Open publishes all reviews undertaken for accepted manuscripts. Reviewers are asked to complete a checklist review form and are provided with free text boxes to elaborate on their assessment. These free text comments are reproduced below.

ARTICLE DETAILS

Title (Provisional)

Does Hourly Variation in Ambient Nitrogen Dioxide Increase the Risk of Acute Asthma Hospitalisations? A Time-Series Study of Birmingham and Solihull, UK

Authors

Shukla, Nidhi; Kwok, Alan; Topham, Alexander; Gallier, Suzy; Sapey, Elizabeth; Percy, Andrew; Bayliss, Mark; Nagakumar, Prasad; Anna L, Hansell; Bartington, Suzanne; Lucas, Tim CD

VERSION 1 - REVIEW

**Reviewer**	**1**
**Name**	**Couillard, Simon**
**Affiliation**	**University of Oxford, Oxford Respiratory NIHR BRC, Nuffield Department of Medicine**
**Date**	**01-Dec-2025**
**COI**	**None**

Thank you for your interesting Birmingham-based analysis of the assocation between NO2 exposure and asthma exacerbation risk. The paper is well written, robustly described from a methodological and results perspective. The discussion is well-balanced.

My comments are minor:

1. Table 2, interesting results. It is unclear to my sense if you tested interaction (NO2 * covariate) or subgroup analyses within the NO2-exposed. Clarification would be needed. My impression (and most of my interest) would be for interaction testing, i.e.: are some covariates compounding the prognostic effects of NO2. Clarification of the language on the subject would be helpful in the methods, results, and discussion sections.

2. To my sense, the fact that sociodemographic features interact positively with NO2 would be highly interesting, and worth mentioning in the abstract (results and conclusion) and paper concluding statements (1st and last paragraph of discussion). This is a suggestion, I leave it to the authors to decide.

3. Figure 3, please shrink by 80% the height of the density plot, which for currently seems more important than the spline. Please also remove Y-axis ticks, as these are not clinically relevant.

4. Discussion, worth highlighting the relevant importance of NO2 on exacerbation risk. You can reference to the helpful quantitative estimates of Meulmeester et al Lancet Respiratory medicine 2025:

- NO2 exposures may carry greater risk than a 10% decrement in baseline FEV1 or a 0.5 increase in ACQ!

Great paper, thanks for doing this.

**Reviewer**	**2**
**Name**	**Shamsutdinova, Diana**
**Affiliation**	**King's College London, Biostatistics and Health Informatics**
**Date**	**15-Apr-2026**
**COI**	**None**

Thank you for allowing me to review this well-designed association study using DLNM and rich temporal data. I have raised several methodological and conceptual issues to be addressed before this can proceed to publication, including clear separation of association vs prediction; explicit treatment of overdispersion (quasi-Poisson sensitivity analysis); improved clarity about the implications of conservative modelling choices; substantial editorial and methodological clarifications.

I appreciate the opportunity to review this manuscript. I found the overall modelling framework (time-series Poisson generalized additive model with DLNM) is appropriate for assessing short-term associations between air pollution and hourly rates of asthma hospital admissions; the model specification is sufficiently flexible without being excessively parametrised, reducing overfitting risk.

However, there are several conceptual and methodological issues that need to be addressed:

1) The use of the term “predicts” in the title is not justified and wrong. The current analysis is a time-series association study, with no predictive modelling techniques. No formal assessment of predictive performance is presented, such as validation on hold-out data, cross-validation, assessment of out-of-sample error. Further, the predictive power of the NO2 is never assessed, e.g. by evaluating marginal predictive contribution of NO_2 by model comparison, or by any other feature importance metric. Also, in the discussion and conclusion, association is rightly mentioned. An expected title should rather be “Short-term longitudinal associations between hourly nitrogen dioxide (NO_2) concentrations and emergency hospital admissions for acute asthma.” or similar.
2) Overdispersion and variance correction can also be an important methodological issue. It is likely that many hourly rates contain 0 asthma admissions, and a Poisson distribution, that

implies equal mean and variance, is a poor fit, and leads to biased confidence intervals (too narrow and overly optimistic). There is no reported assessment of the overdispersion in the manuscript. A way around is fitting quasi-Poisson models (in R GAM) rather than Poisson. Given the number of analyses the authors did, it may be faster to perform a sensitivity analysis of the final model using variance correction and check if the results stand.

3) On the other hand, some modelling decisions seem conservative in the analysis (resulting in lower association estimates). I do not have particular issues with those, but their implications should be made explicit to the reader, and some sensitivity analysis may also be helpful to support the study findings:
    a. Adjusting for PM2.5 in NO2 association model: those pollutants maybe correlated and share traffic-related variations. Hence, it should also be clarified that the estimated NO_2 effects represent associations independent of PM2.5 concentrations. It is also a strength of the study, given that they referenced a similar study for PM2.5. The authors may also consider presenting sensitivity analyses without PM2.5 adjustment to show how effect estimates change.
    b. Adjusting for hour of day. I can agree with the authors' choice of including day hours to the model, however, they should clarify that their model therefore investigates "whether the NO_2 variation above or below typical levels for a given hour associate with increased asthma admissions". Without hour-of-day adjustment, the question would be: "Are times of day with higher NO_2 (e.g. traffic peaks) associated with higher asthma admission rates?" In discussion (lines 455-470), the authors contrast their study with those using daily means, stressing the importance of data granularity. Removing hour of day may be a way to reinforce this point, that is, that inter-daily variations in NO2 are associated with hourly asthma admission rates (with some lag). Therefore, a sensitivity analysis excluding hour-of-day adjustment could be highly informative, demonstrating how inter-daily versus intra-hour variation contributes to observed associations.
    c. The stratified analyses by sex, deprivation index, and ethnicity are appropriate and well-motivated. However, terminology can be corrected: these analyses do not estimate distinct "effect sizes for participant groups" (as written in the abstract) but rather assess effect modification of the NO_2–asthma association.

Minor comments:

Abstract:

- "Hourly emergency hospital admissions" should be clarified as "the hourly rate of emergency hospital admissions for acute asthma."
- Replace "effect sizes for participant groups" with clearer language (see above).

Materials and Methods

Exposure data source:

- The source of hourly NO_2 data must be clearly specified in the Methods section, with a concrete data repository, monitoring network, or dataset reference. The citation "DEFRA Air Pollution in the UK 2023/2024" is too vague.
- Health outcome data: numerous typos, please revise for clarity.
- Inclusion criteria: Please clarify whether patients had to be ≥16 years at the time of admission, or at any point during the study period. Also clearly specify the study period for Solihull ED (e.g. whether it differs from 2016–2022).

- The inclusion criterion should be clearly stated as "Admissions with ICD-10 diagnosis codes J45 or J46 (primary or secondary)."
- COVID-19 exclusions: please clarify whether COVID-19 cases were excluded based on ICD-10 code U07 as a primary diagnosis, a secondary diagnosis, or any mention in the medical record.
- Ethnicity: List the original ethnicity categories and explain how they were collapsed into "White" and "Non-White."
- Statements describing patient characteristics (e.g. "The majority of patients were White British") belong in the Results section and should include percentages.
- "As Solihull does not have an urban background monitor, monitoring records from Birmingham were used to estimate exposure for patients residing in Solihull postcode areas. " – please elaborate how and by whom was this done (by the authors of this study, or by the primarily environmental data collectors and published with the estimates already)

Exposure assessment

- "Night-time characteristics differ in terms of admission patterns, as asthma severity, patient behavior, sleep pattern and more difficult access to healthcare causing admission patterns during the day and night to be fundamentally different. " - supporting citation is needed.
- Also, if a triggering exposure happened in the evening, then 3-6h lag would lead to late-night admissions that were excluded in this study. This can be clarified and discussed. Was this done to explicitly remove many 0 hours? - This may be fine but needs clarification.
- "the model controlled for the Stringency Index (SI)." Please clarify how the SI is defined and provide a correspondent reference.
- Appreciate the use of 2 baseline references for NO2 (0 and 7.3 mg/m^3), as 0 looks hypothetical rather than realistic.

VERSION 1 - AUTHOR RESPONSE

Reviewer: 1

Minor Comments:

1. Table 2, interesting results. It is unclear to my sense if you tested interaction (NO2 * covariate) or subgroup analyses within the NO2-exposed. Clarification would be needed. My impression (and most of my interest) would be for interaction testing, i.e.: are some covariates compounding the prognostic effects of NO2. Clarification of the language on the subject would be helpful in the methods, results, and discussion sections.

RESPONSE: Thank you for this helpful comment. We agree that interaction testing would provide a more rigorous assessment of effect modification. However, we would like to clarify that, similar to many time-series studies, our analysis did not include formal interaction terms (NO_2 × covariate). Instead, we performed stratified (subgroup) analyses to explore whether the association between NO_2 and the outcome differed across these groups. The clarification has been added to **Lines 271-272 and 376-379.**

2. To my sense, the fact that sociodemographic features interact positively with NO2 would be

highly interesting, and worth mentioning in the abstract (results and conclusion) and paper concluding statements (1st and last paragraph of discussion). This is a suggestion, I leave it to the authors to decide.

RESPONSE: Thank you for this comment, and we agree that this is an important finding that should be given greater prominence in the manuscript. We have amended accordingly, and the following lines have been added to the abstract and the discussion 1st paragraph.
Lines 42-44: Furthermore, subgroup-specific analysis indicates a higher positive risk associated with hourly NO_2 among patients living in the most deprived areas.
Line 423-425: Furthermore, subgroup analysis indicated a significant positive association between hourly NO_2 exposure and the risk of hospital admissions among patients living in the most deprived areas.

3. Figure 3, please shrink by 80% the height of the density plot, which for currently seems more important than the spline. Please also remove Y-axis ticks, as these are not clinically relevant.

RESPONSE: Thank you for this suggestion. The new version of the manuscript includes the revised Figure 3 as suggested.

4. Discussion, worth highlighting the relevant importance of NO2 on exacerbation risk. You can reference to the helpful quantitative estimates of Meulmeester et al., Lancet Respiratory medicine 2025:

- NO2 exposures may carry greater risk than a 10% decrement in baseline FEV1 or a 0.5 increase in ACQ!

RESPONSE: Thank you for this suggestion. We highlight that Meulmeester et al. 2025, quantified key clinical predictors of severe asthma attacks: a 0.5 increase in ACQ-5 score corresponds to an RR of 1.10 (95% CI 1.07–1.13), and a 10% decrement in baseline FEV_1% predicted corresponds to an RR of 1.11 (95% CI 1.08–1.15), which is lower than the risk of exacerbation associated with NO_2 as observed in this analysis. While the study did not directly assess the comparison of these predictors and NO_2 exposure. Therefore, we suggested future studies should directly compare the magnitude of NO_2 exposure risk against changes in baseline FEV_1 and ACQ scores to clarify their relative contributions to exacerbation risk. Modification added in **lines 556-563.**

Reviewer: 2

I appreciate the opportunity to review this manuscript. I found the overall modelling framework (time-series Poisson generalized additive model with DLNM) is appropriate for assessing short-term associations between air pollution and hourly rates of asthma hospital admissions; the model specification is sufficiently flexible without being excessively parametrised, reducing overfitting risk.

However, there are several conceptual and methodological issues that need to be addressed:

1. 1) The use of the term "predicts" in the title is not justified and wrong. The current analysis is a

   time-series association study, with no predictive modelling techniques. No formal assessment of predictive performance is presented, such as validation on hold-out data, cross-validation, assessment of out-of-sample error. Further, the predictive power of the NO2 is never assessed, e.g. by evaluating marginal predictive contribution of NO2 by model comparison, or by any other feature importance metric. Also, in the discussion and conclusion, association is rightly mentioned. An expected title should rather be "Short-term longitudinal associations between hourly nitrogen dioxide (NO2) concentrations and emergency hospital admissions for acute asthma." or similar.

RESPONSE: We agree that this analysis does not include predictive techniques and have modified the title accordingly.

New title: "Short-term associations between hourly nitrogen dioxide (NO_2) concentrations and emergency hospital admissions for acute asthma."

2. 2) Overdispersion and variance correction can also be an important methodological issue. It is likely that many hourly rates contain 0 asthma admissions, and a Poisson distribution, that implies equal mean and variance, is a poor fit, and leads to biased confidence intervals (too narrow and overly optimistic). There is no reported assessment of the overdispersion in the manuscript. A way around is fitting quasi-Poisson models (in R GAM) rather than Poisson. Given the number of analyses the authors did, it may be faster to perform a sensitivity analysis of the final model using variance correction and check if the results stand.

   RESPONSE: Thank you for the suggestion. The dispersion parameter for the quasi-Poisson model was 1.0058. Therefore, the Poisson model was used in the analysis. The cumulative risk plot comparing the Poisson and Quasi-Poisson models is added to the supplementary material (Figure S5) of this manuscript.

3. 3) On the other hand, some modelling decisions seem conservative in the analysis (resulting in lower association estimates). I do not have particular issues with those, but their implications should be made explicit to the reader, and some sensitivity analysis may also be helpful to support the study findings:

   1. Adjusting for PM2.5 in NO2 association model: those pollutants maybe correlated and share traffic-related variations. Hence, it should also be clarified that the estimated NO2 effects represent associations independent of PM2.5 concentrations. It is also a strength of the study, given that they referenced a similar study for PM2.5. The authors may also consider presenting sensitivity analyses without PM2.5 adjustment to show how effect estimates change.

      RESPONSE: Thank you for your comments. Adding further sensitivity analyses results would definitely strengthen the insights of this manuscript. We

agree that NO_2 and $PM_{2.5}$ share traffic-related variation. Accordingly, all primary NO_2 estimates are derived from models adjusted for $PM_{2.5}$ and therefore represent associations independent of $PM_{2.5}$ concentrations. We have added a sensitivity analysis plot to the supplementary material (Figure S6) showing that the model with and without $PM_{2.5}$ adjustment has nearly identical relative risks (RRs).

2. Adjusting for hour of day. I can agree with the authors' choice of including day hours to the model, however, they should clarify that their model therefore investigates "whether the NO2 variation above or below typical levels for a given hour associate with increased asthma admissions". Without hour-of-day adjustment, the question would be: "Are times of day with higher NO2 (e.g. traffic peaks) associated with higher asthma admission rates?" In discussion (lines 455-470), the authors contrast their study with those using daily means, stressing the importance of data granularity. Removing hour of day may be a way to reinforce this point, that is, that inter-daily variations in NO2 are associated with hourly asthma admission rates (with some lag). Therefore, a sensitivity analysis excluding hour-of-day adjustment could be highly informative, demonstrating how inter-daily versus intra-hour variation contributes to observed associations.

   RESPONSE: We thank the reviewer for this insightful suggestion. The intention to add the hour-of-day in the model is to ensure that observed associations are not merely artifacts of routine weekly or hourly patterns, but instead reflect actual relationships between NO2 concentration and hourly hospital admissions [line 243-251]. Further clarification is added to the manuscript as suggested by the reviewer in lines 248-250. In addition, the sensitivity analysis is added in the supplementary material (Figure S6). The removal of the hour-of-day showed a slight increase in cumulative risk of admission.

3. The stratified analyses by sex, deprivation index, and ethnicity are appropriate and well-motivated. However, terminology can be corrected: these analyses do not estimate distinct "effect sizes for participant groups" (as written in the abstract) but rather assess effect modification of the NO2–asthma association.

   RESPONSE: This is corrected at relevant places as per the recommendation.

Minor comments:

Abstract:

- "Hourly emergency hospital admissions" should be clarified as "the hourly rate of emergency hospital admissions for acute asthma."

  RESPONSE:. The statement is corrected as suggested.

- Replace "effect sizes for participant groups" with clearer language (see above).

RESPONSE: The statement is corrected as suggested.

Materials and Methods

Exposure data source:

- The source of hourly NO2 data must be clearly specified in the Methods section, with a concrete data repository, monitoring network, or dataset reference. The citation "DEFRA Air Pollution in the UK 2023/2024" is too vague.

  RESPONSE: We apologise for the confusion. However, as mentioned in the method section between lines 199-204, "The environmental data, such as hourly NO_2, were obtained from urban background monitors in the Defra-managed Automatic Urban and Rural Network (AURN), which provides an integrated contribution from all sources upwind of the station. The study includes three urban background monitors located in Birmingham (UKA00559, UKA00655, and UKA00479), as shown in Figure 1. The data from these monitors was downloaded from the publicly available Defra platform." As a reference, the URL to the Defra website for this data is included for clarity, and the previous reference is removed. Furthermore, the statement is revised for more clarity.

Health outcome data: numerous typos, please revise for clarity.

- Inclusion criteria: Please clarify whether patients had to be ≥16 years at the time of admission, or at any point during the study period. Also clearly specify the study period for Solihull ED (e.g. whether it differs from 2016–2022).

  RESPONSE: The age is at the time of admission; clarified in the manuscript line 164. The study period for patients from Solihull was from 1 June 2016 to 30 April 2020; Line 171-172 is added for more clarity.

- The inclusion criterion should be clearly stated as "Admissions with ICD-10 diagnosis codes J45 or J46 (primary or secondary)."

  RESPONSE: It is corrected as suggested.

- COVID-19 exclusions: please clarify whether COVID-19 cases were excluded based on ICD-10 code U07 as a primary diagnosis, a secondary diagnosis, or any mention in the medical record.

  RESPONSE: The data include only patients with U07.1 and U07.2 code. All cases with confirmed COVID-19 by laboratory testing (U07.1) and inconclusive COVID-19 cases (U07.2; virus not identified), based on either the primary or secondary diagnostics, were excluded from the analysis.

- Ethnicity: List the original ethnicity categories and explain how they were collapsed into "White" and "Non-White."

  RESPONSE: The list of ethnic group categories is provided in the supplementary material (Table S6). An explanation has been added to the manuscript: Line 190-194

"The majority of patients were of White ethnic background (including White British, White Irish, and other White groups), of whom 97% were White British. Non-White patients include all other ethnic categories, including mixed ethnicities. The patients under the "not-known" ethnicity category were excluded from the analysis."

- Statements describing patient characteristics (e.g. "The majority of patients were White British") belong in the Results section and should include percentages.

RESPONSE: This is corrected as a part of the previous comment.

- "As Solihull does not have an urban background monitor, monitoring records from Birmingham were used to estimate exposure for patients residing in Solihull postcode areas. " – please elaborate how and by whom was this done (by the authors of this study, or by the primarily environmental data collectors and published with the estimates already)

RESPONSE:. The decision was made collectively by the authors. However, we agree that this is a limitation and might have caused some misclassification. The limitation is discussed in the manuscript.

Exposure assessment

- "Night-time characteristics differ in terms of admission patterns, as asthma severity, patient behaviour, sleep pattern and more difficult access to healthcare, causing admission patterns during the day and night to be fundamentally different. " - supporting citation is needed.

RESPONSE: As suggested, the required reference is added on line 213.

- Also, if a triggering exposure happened in the evening, then 3-6h lag would lead to late-night admissions that were excluded in this study. This can be clarified and discussed. Was this done to explicitly remove many 0 hours? - This may be fine but needs clarification.

RESPONSE: Thanks for this important comment. Evening admissions were primarily excluded because admission characteristics differ in terms of admission patterns, such as asthma severity, patient behaviour, sleep patterns, and more difficult access to healthcare, causing admission patterns during the day and night to be fundamentally different. For instance, while people are asleep, they cannot attend the hospital unless they get woken up by their asthma. Additionally, people probably try to avoid attending the hospital at night. Therefore, overall, the pathway to admission is very different at night. However, we agree that exposures occurring during the evening may contribute to hospital admissions within the subsequent 3–6 hours, which could include late-night admissions excluded from the analysis. We have now clarified this and added further discussion in the manuscript. The manuscript discusses this issue in paragraph between line 520-527. Further, the Lines 523-525 were added; "However, exposures occurring during the evening may contribute to admissions within the subsequent 3–6 hours,

potentially leading to late-night admissions that were not captured in the present analysis”

- "the model controlled for the Stringency Index (SI)." Please clarify how the SI is defined and provide a correspondent reference.

  RESPONSE: Thanks for this comment, and apologies for missing this reference. We have now clarified the definition of the Stringency Index (SI) in the manuscript and added the corresponding reference. Lines 257-264: “The SI was obtained from the Oxford Coronavirus Government Response Tracker (OxCGRT) project and represents a composite measure of nine government response indicators, including school closures, workplace closures, cancellation of public events, restrictions on gatherings and movement, stay-at-home requirements, public information campaigns, and international travel controls. The index ranges from 0 to 100, with higher values indicating stricter governmental responses.”

  “Hale T, Angrist N, Goldszmidt R, et al. A global panel database of pandemic policies (Oxford COVID-19 Government Response Tracker). Nature human behaviour 2021;5(4):529-38.”

- Appreciate the use of 2 baseline references for NO2 (0 and 7.3 mg/m^3), as 0 looks hypothetical rather than realistic.

  RESPONSE: Thank you for appreciating. The reference 0 was used to compare the effect in the absence of exposure.

VERSION 2 - REVIEW

**Reviewer**	**1**
**Name**	**Couillard, Simon**
**Affiliation**	**University of Oxford, Oxford Respiratory NIHR BRC, Nuffield Department of Medicine**
**Date**	**31-May-2026**
**COI**	

Thank you. No further comment except a note : figure 3 has a lot of wasted space (y axis up to 1.0), could cut the axis at time of final submission.

**Reviewer**	**2**
**Name**	**Shamsutdinova, Diana**

Affiliation King's College London, Biostatistics and Health Informatics

Date 25-Jun-2026

COI

Thank you for sending the revised version of the paper. The manuscript has substantially improved after the revision and my comments were adequately addressed, I do not have any further comments.

VERSION 2 - AUTHOR RESPONSE

Reviewer: 1
Dr. Simon Couillard, University of Oxford, Université de Sherbrooke
Comments to the Author:
Thank you. No further comment except a note : figure 3 has a lot of wasted space (y axis up to 1.0), could cut the axis at time of final submission.

Thank you for your thoughtful comments. The data used in our analysis were drawn from a fixed-term contract that has now ended, and the original datasets and plotting scripts are no longer accessible. Because of these constraints, we are unable to change the axis scale at this stage. We do not believe an axis change would alter the substantive findings or add meaningful information to the manuscript. We hope the explanation is acceptable and are grateful for the reviewer’s careful reading.

Reviewer: 2
Dr. Diana Shamsutdinova, King's College London
Comments to the Author:
Thank you for sending the revised version of the paper. The manuscript has substantially improved after the revision and my comments were adequately addressed, I do not have any further comments.

Thank you for your comments and encouragement.
